# Rab33B Controls Hepatitis B Virus Assembly by Regulating Core Membrane Association and Nucleocapsid Processing

**DOI:** 10.3390/v9060157

**Published:** 2017-06-21

**Authors:** Christina Bartusch, Tatjana Döring, Reinhild Prange

**Affiliations:** Department of Medical Microbiology and Hygiene, University Medical Center of the Johannes Gutenberg University Mainz, Augustusplatz, D-55131 Mainz, Germany; christina.bartusch@uni-mainz.de (C.B.); doeringt@uni-mainz.de (T.D.)

**Keywords:** hepatitis B virus, Rab GTPase, Rab33B, core/capsid membrane association, nucleocapsid assembly, virus trafficking

## Abstract

Many viruses take advantage of cellular trafficking machineries to assemble and release new infectious particles. Using RNA interference (RNAi), we demonstrate that the Golgi/autophagosome-associated Rab33B is required for hepatitis B virus (HBV) propagation in hepatoma cell lines. While Rab33B is dispensable for the secretion of HBV subviral envelope particles, its knockdown reduced the virus yield to 20% and inhibited nucleocapsid (NC) formation and/or NC trafficking. The overexpression of a GDP-restricted Rab33B mutant phenocopied the effect of deficit Rab33B, indicating that Rab33B-specific effector proteins may be involved. Moreover, we found that HBV replication enhanced Rab33B expression. By analyzing HBV infection cycle steps, we identified a hitherto unknown membrane targeting module in the highly basic C-terminal domain of the NC-forming core protein. Rab33B inactivation reduced core membrane association, suggesting that membrane platforms participate in HBV assembly reactions. Biochemical and immunofluorescence analyses provided further hints that the viral core, rather than the envelope, is the main target for Rab33B intervention. Rab33B-deficiency reduced core protein levels without affecting viral transcription and hampered core/NC sorting to envelope-positive, intracellular compartments. Together, these results indicate that Rab33B is an important player in intracellular HBV trafficking events, guiding core transport to NC assembly sites and/or NC transport to budding sites.

## 1. Introduction

The hepatitis B virus (HBV) is one of the smallest animal viruses, yet one of the most successful human pathogens. The virus chronically infects about 350 million people worldwide and can ultimately lead to liver failure and liver cancer. HBV infection remains an important public health threat, as current therapeutics can suppress, but cannot cure infection. Due to the tiny size of its genome with about 3 kb and four open reading frames, HBV likely depends on a close interplay between viral and host factors for the generation of new viral particles from infected cells.

HBV is an enveloped DNA virus that replicates via reverse transcription and exclusively infects human hepatocytes by using a bile-acid transporter, the sodium taurocholate co-transporting polypeptide (NTCP), as a receptor [1]. After virus entry and disassembly, the nucleocapsids (NC) are delivered to the nucleus where the partially double-stranded DNA (dsDNA) genome is converted to the covalently closed circular DNA (cccDNA). The episomal cccDNA serves as a template for the transcription of the pregenomic RNA (pgRNA) and the viral mRNAs [2,3]. Following nuclear export and protein synthesis, the assembly of progeny virions begins with the formation of the icosahedral NC that packages the viral pgRNA together with the covalently linked viral polymerase (Pol). The capsid is formed by 120 dimers of the single core protein, encompassing 183 or 185 amino acids (aa), depending on the genotype [4,5]. The core protein consists of two separate domains: the N-terminal domain, which is sufficient to form the capsid shell, and the C-terminal domain (CTD), which is dispensable for capsid assembly but essential for viral replication. The CTD is highly basic and dynamically phosphorylated, which is important for the encapsidation of the pgRNA/Pol complex, DNA synthesis, and nuclear targeting. Inside the immature NC, the pgRNA is reverse transcribed to the dsDNA genome via a single-stranded DNA intermediate [2,3]. Mature NC, formed in the cytoplasm, can then be enclosed by the viral envelope composed of cellular lipids and three related viral glycoproteins, the small S, middle M, and large L envelope proteins that originate at the endoplasmic reticulum (ER) membrane [5,6]. Alternatively, mature NC can deliver the dsDNA genome to the nucleus for cccDNA amplification via an active intracytoplasmic transport along microtubules [7].

How NCs move to budding sites at intracellular membranes is less understood. HBV budding requires functions of the endosomal sorting complex required for transport (ESCRT) machinery [8,9,10,11]. This network is normally involved in the formation of vesicles that bud away from the cytosol into the lumen of multivesicular bodies (MVBs). Aside, ESCRT-associated proteins, like the ubiquitin-interacting adaptor γ2-adaptin [12,13], the Nedd4 ubiquitin ligase [12], and α-taxilin [14] have been shown to be important for HBV budding, likely via escorting the viral subunits to the ESCRT machinery. 

Beside infectious virions, HBV produces and releases subviral empty envelope particles (SVPs) and subviral non-enveloped capsid/NC particles [15,16]. SVPs greatly outnumber mature virions and presumably act as decoys for the immune system. They are formed by self-assembly of S and budding into intraluminal cisternae of post-ER/pre-Golgi compartments and exit the cell via the constitutive secretory pathway [15,17]. The pathway of release of naked capsids, devoid of a membrane coat, is independent of ESCRT functions but depends on Alix [16], a multifunctional protein with key roles in membrane biology. In addition, this pathway is assisted by the cellular Rab33B GTPase [18]. 

Eukaryotic Rab proteins comprise a large group of small monomeric GTPases that organize membrane platforms, control membrane identity and vesicle budding, trafficking, and fusion. Rab proteins exert their functions through highly regulated GTP-GDP cycles, thereby enabling the GTP-bound, active forms to recruit specific effector proteins onto membranes [19]. Originally, Rab33B was identified as a Golgi-resident protein involved in retrograde Golgi-to-ER transport, but more recent work suggested an involvement of Rab33B in autophagy processes [20,21]. The degrading autophagy pathway involves concerted action of more than 20 specific autophagy (Atg) proteins that mediate the formation and elongation of a double-membrane sack, cargo engulfment, autophagophore closure, and autophagosome fusion with the lysosome. Autophagophore formation is initiated by the ternary Atg5/12/16L1 complex that can interact with catalytic active Rab33B, likely to regulate autophagophore expansion. To do so, Rab33B-decorated vesicles derived from the Golgi may bind to Atg5/12/16L1 and fuse with the elongating autophagophore for membrane supply [20,22].

Previously, we showed that HBV naked capsid formation and egress require Rab33B in conjunction with its Atg5/12/16L1 effector, while a complete and functional autophagy pathway is dispensable [18]. Although the precise mechanistic insights are still lacking, Rab33B and Atg5/12/16L1 together with autophagic membrane platforms may provide a scaffold for HBV naked capsid maturation and assembly. Simultaneously, these host structures may provide a mean to deliver naked capsids to the extracellular milieu without lysing the cell. Aside, the autophagic machinery has been implicated in HBV virion replication both morphologically and functionally, as targeted knockdowns of the autophagic proteins Atg5, Atg7, or Beclin1 reduce HBV release [23,24,25,26]. The processes by which HBV utilizes these organelles to its own end are less defined.

In order to unravel the interplay between HBV and the Rab GTPase network, we here analyzed the impact of Rab33B in HBV trafficking and exocytosis using HBV-replicating liver cell lines. Thus far, two Rab GTPases, Rab5 and Rab7, have been described to control HBV infection [27]. During virus entry and uptake, Rab5 and Rab7 have been shown to act as dependency factors [27]. Conversely, in the late steps of HBV replication, Rab7 functions as a restriction factor, as the depletion of Rab7 increased HBV secretion, likely as a consequence of reduced delivery of the virus to the degrading lysosome [28]. In extension, we here showed that HBV usurps Rab33B-guided cell pathways for its own benefit.

## 2. Materials and Methods 

### 2.1. Expression Constructs 

For HBV replication, the episomal expression vector pCEP4∆CMV∆SV40/1.1xHBV (pHBV) was used. It carries a 1.1× unit length HBV genome (genotype D; GenBank™ accession number J02203) in which the viral core/Pol promoter is preceded by the human metallothionein (hMT) IIA promoter. The pCEP vector backbone was modified such that the cytomegalovirus (CMV) promoter and the Simian virus 40 (SV40) polyadenylation signal were deleted [10]. For HBV transcription analyses, a modified version of the HBV replicon was engineered (pHBV∆HP) in which the foreign hMT promoter region was removed [10]. The plasmid pHBV∆Env, defective in L, M, and S envelope protein expression, had been described [29]. For solitary expression of the HBV S envelope protein, plasmid pNI2.S.HA was employed, which carries the S gene with a C-terminally tagged influenza virus hemagglutinin (HA) epitope under the control of the hMT promoter [12]. The vector pNI2.C contains the HBV core gene (without the pre-core region) preceded by the hMT promoter, as described [16]. The core mutant Core∆CTD, carrying a deletion of aa 145 to 183, was created by cloning. The plasmid pEGFP.Rab33B harbors the mouse Rab33B gene fused to the C-terminus of the enhanced green fluorescent protein (EGFP) and was kindly provided by Mitsunori Fukuda (Tohoku University, Sendai, Japan). The constitutive dominant negative Rab33B.dn mutant carries a threonine-to-asparagine-substitution at aa position 47 [20]. For small interfering (siRNA) rescue experiments, we constructed an attenuated protein expression vector carrying a truncation within the CMV promoter region (pCMV∆5), essentially as described by Morita et al. [30]. Therefore, the CMV promoter present in plasmid pCMV.Myc (Clontech, Mountain View, CA, USA) was deleted for nucleotides 28 to 459 by mutagenesis using the Q5^®^ Site-Directed Mutagenesis Kit (New England Biolabs, Ipswich, MA, USA). The mouse Rab33B gene was excised from pEGFP.Rab33B and cloned into the rescue vector rendering an N-terminally Myc-tagged Rab33B construct. 

### 2.2. siRNAs, Cell Culture, and Transfection

The human hepatocellular carcinoma cell line HuH-7 was obtained from the Japanese Collection of Research Bioresources (http://cellbank.nibiohn.go.jp/english/). The HepG2 cell line was bought from the American Type Culture Collection (https://www.atcc.org/) and the HepG2215 cell line, which persistently produces HBV due to the integrated HBV genome, was kindly provided by Patrick Gerner (University Medical Center, Mainz, Germany). Cells were cultured in Dulbecco’s modified Eagle’s medium (ThermoFisher Scientific, Waltham, MA, USA) supplemented with 10% fetal bovine serum and 5 µg/mL ciprofloxacin (Fresenius Kabi, Bad Homburg, Germany). Transfections of HuH-7 cells with plasmid DNAs were performed with Lipofectamine^®^ Plus (ThermoFisher Scientific, Waltham, MA, USA). Unless otherwise indicated, 2 × 10^5^ cells per well of a six-well plate were transfected with a final concentration of 4 µg plasmid DNA. For transfection of HuH-7 cells with siRNAs without plasmid DNA, the Lipofectamine^®^ RNAiMAX transfection reagent (ThermoFisher Scientific) was used. Briefly, 1.3 × 10^5^ cells per well of a six-well plate were transfected with 20 pmol siRNA according to the protocol of the supplier. For silencing of human Rab33B, a pool of four siRNAs obtained from Dharmacon (Lafayette, CO, USA) was used (5′-CACAAACCAUUAAUGCUUA-3′, 5′-GAUAGAAGAAUGCAAACAA-3′, 5′-GAUAUACCACGGAUUCUUG-3′, 5′-GGUGGAUUUCCGAGAACGA-3′). As a control, a nonsense siRNA with no known homology to mammalian genes was used (Qiagen, Hilden, Germany). After 48 h, cells were retransfected with plasmid DNA using Lipofectamine^®^ Plus and were harvested after an additional 48 to 72 h.

### 2.3. Antibodies

Polyclonal antisera against recombinant native (K45) or denatured (K46) core particles were raised in rabbits, as described [12]. In addition, commercially available polyclonal rabbit (#B0586; Dako, Carpinteria, CA, USA) or monoclonal mouse (#3HB17; HyTest, Turku, Finland) antisera against the core antigen or capsid particle, respectively, were used. For immunodetection of the L protein, either the MA18/7 mouse antibody (a gift from Dieter Glebe, University of Gießen, Gießen, Germany) or a rabbit antibody raised against a recombinant peptide encoding aa 1–42 of L fused to glutathione *S*-transferase (GST) (#K1350; Eurogentec, Liège, Belgium) were employed. Other commercially available antibodies were as follows: rabbit anti-Atg5 (#D5F5U; Cell Signaling, Danvers, MA, US), rabbit anti Atg16L1 (#D6D5; Cell Signaling, Danvers, MA, USA); rabbit anti-calnexin (#SPA-860; Enzo Life Sciences, Farmingdale, NY, USA)); mouse anti-GFP (#JL-8; Clontech, Mountain View, CA, USA); mouse anti-golgin-97 (#A-21270; Molecular Probes, Eugene, OR, USA); mouse anti-HA (#16B12; Covance, HISS Diagnostics, Freiburg im Breisgau, Germany); mouse anti-β-actin (#AC15; Sigma-Aldrich, St. Louis, MO, USA), mouse anti-estrogen receptor binding site associated antigen 9 (EBAG9, #4A10; Sigma-Aldrich), mouse anti-Rab33B (#6F4; Sigma-Aldrich), α-tubulin (#B-5-1-2; Sigma-Aldrich, St. Louis, MO, USA); and mouse anti-Rab33A/B (#sc-271199; Santa Cruz, CA, USA), rabbit anti-protein disulfide isomerase (PDI, #sc-20132; Santa Cruz). Peroxidase-labeled, secondary antibodies were obtained from Dianova (Hamburg, Germany), and fluorophor-labeled antibodies were purchased from Molecular Probes.

### 2.4. Subviral Particle Analysis

Cells were lysed with 1× Laemmli buffer (50 mM Tris-HCl pH 6.8, 10% β-mercaptoethanol, 10% glycerol, 2% sodium dodecyl sulfate (SDS), 0,1% bromphenol blue), scraped from the plates and boiled for 10 min prior to centrifugation for 5 min at 13,000× *g* and 4 °C. To analyze the release of SVPs, clarified culture medium was concentrated by ultracentrifugation through a 20% (*w*/*v*) sucrose cushion (4 h at 100,000× *g* and 4 °C). Pellets were suspended in 1× Laemmli buffer. Lysates and concentrated supernatants were subjected to sodium dodecyl sulfate–polyacrylamide gel electrophoresis (SDS–PAGE) and Western blotting (WB) analyses using standard procedures.

### 2.5. Cell Protein Analyses, Viral Particle Analysis, and Multiplex Real-Time PCR

The production of HBV particles was determined by a TaqManchemistry-based, multiplex real-time PCR, as described [10]. Cells were lysed with 50 mM Tris-HCl pH 7.5, 150 mM NaCl, 5 mM MgCl_2_, and 0.2% Triton X-100, and lysates were centrifuged as outlined above. To probe for protein expression, aliquots of the lysates were analyzed by WB. Intracellular NC and extracellular virions were isolated by immunomagnetic separation using PureProteome Protein G Magnetic Beads (Millipore, Billerica, MA, USA) coated with capsid- and envelope-specific antibodies, respectively [10]. After isolation of the viral DNA, PCR analyses were performed with a 7300 Real-Time PCR System and Sequence Detection Software 4.0 (Applied Biosystems, Foster, CA, USA). Because the transfected HBV replicon plasmid DNA (pHBV) and progeny virus DNA are genetically identical, two primer/probe sets were used to target either the HBV genome or the hygromycin resistance gene of the pCEP plasmid backbone, as described [10]. In parallel, lysates and supernatants of transfected cells were assayed by ELISAs. To probe for the synthesis of the HBV envelope protein, hepatitis B surface antigen (HBsAg) reactivity was determined with the Murex HBsAg Version 3 kit (Abbott, Chicago, IL, USA). The biosynthesis of the HBV pre-core/core protein was assayed with the ETI-EBK PLUS ELISA kit (DiaSorin, Saluggia, Italy) as instructed by the supplier. To evaluate the presence of damage and toxicity of the transfected cells, lactate dehydrogenase (LDH) activity was determined in culture media using a colorimetric quantification assay (Cytotoxicity Detection Kit^PLUS^; Roche Diagnostics, Rotkreuz, Switzerland). To measure total protein concentrations in cell extracts, a Bradford protein assay (Bio-Rad, Hercules, CA, USA) with bovine serum albumin standards (New England Biolabs, Ipswich, MA, USA) was used.

### 2.6. Quantitative Reverse Transcription-PCR Analysis

Total mRNAs were isolated from cells using the TRIzol reagent (Life Technologies, Carlsbad, CA, USA) and the Direct-zol^™^ RNA MiniPrep kit (Zymo Research, Irvine, CA, USA), according to the protocols of the suppliers. The mRNA was treated with 5 U RNase-free, recombinant DNase I (Roche Diagnostics), and cDNA synthesis was performed using the qScript cDNA Synthesis Kit (Quanta Biosciences, Beverly, MA, USA). For reverse transcription (RT-)PCR, each reaction mixture (20 µL) contained 5 µL cDNA template, 1 µL forward primer (10 µM), 1 µL reverse primer (10 µM), 10 µLFast Start Universal SYBR Green Master (Roche Diagnostics), and 3 µL aqua bidest. For data analysis, the comparative cycle threshold method (C*_T_*) was used, and data were reported as the fold change normalized to an endogenous reference gene (β-actin). The sense 5′-GGGTGGATTTCCGAGAACGAG-3′ and anti-sense primers 5′-GCTGAACCATGCTCTTTCTGA-3′ were used for Rab33B mRNA detection. For HBV transcription analyses, the sense 5′-TGTCCTCCAACTTGTCCTGGTT-3′ and anti-sense 5′-AGGCATAGCAGCAGGATGAAGA-3′ primers were used that target the 3.5 kb pgRNA, the 3.4-kb pre-core-, the 2.4 and 2.1 kb envelope-specific transcripts, but not the 0.7 kb X-specific mRNA.

### 2.7. Analysis of Soluble and Insoluble Protein Fractions

Transfected HuH-7 cells were lysed for 30 min on ice with radioimmune precipitation buffer (RIPA; 10 mM Tris-HCl pH 7.5, 150 mM NaCl, 1 mM ethylenediaminetetraacetic acid [EDTA], 1% Nonidet P-40, 0.5% deoxycholate, 0.1% SDS) supplemented with the protease inhibitor cocktail (Roche). Soluble and insoluble protein fractions were separated by centrifugation at 13,000× *g* and 4 °C for 15 min. Pellets were resuspended in 50 µL of 10 mM Tris-HCl pH 7.5 and 1% SDS for 10 min at room temperature. After the addition of 200 µL of RIPA buffer, samples were sonicated for 20 s in an ultrasonic bath. 

### 2.8. Fluorescense Microscopy

For immunostaining, cells grown on cover-slips were fixed with 4% paraformaldehyde (PFA) for 10 min at room temperature and permeabilized with 0.2% Triton X-100 for 10 min. Alternatively, cells were fixed and permeabilized with ice-cold methanol containing 2 mM ethylene glycol-bis(β-aminoethyl ether)-*N*,*N*,*N*′,*N*′-tetraacetic acid (EGTA). Cells were blocked in phosphate-buffered saline (PBS) containing 1% bovine serum albumin, incubated with the indicated primary antibodies for 1 h at 37 °C, rinsed with PBS, and then incubated with AlexaFluor-tagged secondary antibodies (Life Technologies, Carlsbad, CA, USA) for 1 h at 37 °C. DNA was stained with Hoechst 33342 (Sigma-Aldrich). Images were acquired separately for each channel using a Zeiss Axiovert 200 M microscope equipped with a Plan-Apochromat 100× (1.4 NA) and a Zeiss Axiocam digital camera (Oberkochen, Germany). Axiovision software 4.7.1 (Zeiss, Oberkochen, Germany) was used for merging pictures. Tiffs were assembled into figures using Adobe Photoshop CS6. 

### 2.9. Membrane Flotation Analysis

For the density flotation assay, cells were broken by dounce homogenization (25 strokes) in homogenization buffer (250 mM sucrose, 1 mM EDTA, 2 mM CaCl_2_, 20 mM HEPES-NaOH, pH 7.4). Extracts were centrifuged at 1000× *g* for 10 min at 4 °C to sediment nuclei and debris. The resulting postnuclear supernatant was adjusted to 40% OptiPrep^TM^ (Sigma-Aldrich) using a 50% OptiPrep solution (*w*/*v*) diluted in 250 mM sucrose, 6 mM EDTA, 12 mM CaCl_2_, 120 mM Hepes-NaOH, pH 7.4 (dilution buffer [DB]; Franklin Lakes, NJ, USA). This fraction (1.5 mL) was placed on the bottom of a SW60 centrifuge tube and overlaid with 30% (1.7 mL) and 5% (0.8 mL) OptiPrep solutions diluted in DB. The step gradient was centrifuged for 3 h at 100,000× *g* and 4 °C in a SW60 rotor (Beckman, Brea, CA, USA), and fractions were collected from the top. To concentrate proteins, fractions were precipitated with TCA prior to SDS–PAGE.

### 2.10. Statistics

Statistical differences between groups and statistical graphs were assessed with a two-tailed, unpaired Student’s *t*-test using Microsoft Office Excel 2016. Differences between groups were considered significant when the *P*-value was * *P* < 0.05, or ** *P* < 0.01.

## 3. Results

### 3.1. Role of Rab33B in HBV SVP Assembly and Release

**Figure 1:**
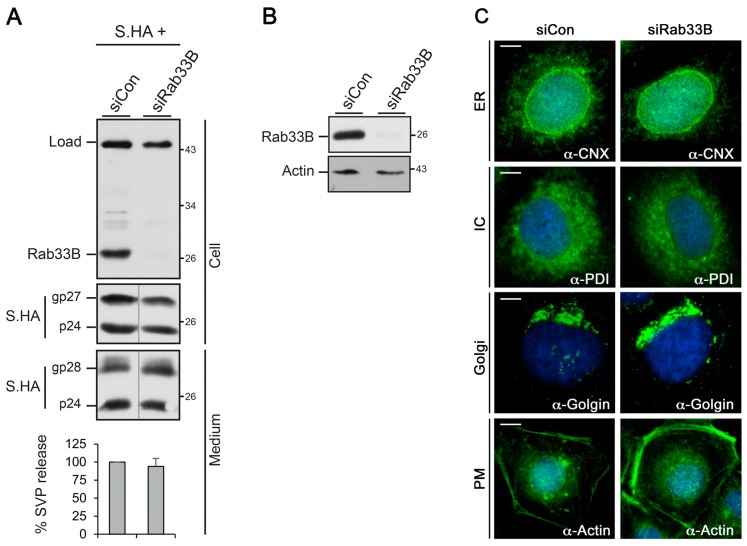
Depletion of Rab33B does not affect hepatitis B virus (HBV) subviral empty envelope particles (SVP) release or cell morphology. (**A**) HuH-7 cells were treated with control small interfering RNA (siCon) or a pool of four siRNA duplexes directed against Rab33B. After 48 h, cells were retransfected with an hemagglutinin (HA)-tagged version of the HBV S envelope gene (S.HA), and lysates and supernatants were harvested 72 h later. To probe for the knockdown efficiency, lysates were immunoblotted with anti-Rab33B antibodies (#6F4). An unspecific band stained by the antibody served as a loading control (Load). Synthesis (Cell) and secretion (Medium) of S.HA were assayed by HA-specific Western blot (WB). The non-glycosylated (p24) and glycosylated forms (gp27, gp28) of S.HA are depicted. Numbers to the right refer to molecular weight standards in kDa. The degree of S.HA release was quantified by densitometric analysis of Western blots and was demonstrated in percent amount relative to the control cells (*n* = 3, mean ± standard deviation [SD]). (**B**) HuH-7 cells were transfected with siCon- or siRab33B-specific duplexes for four days and cell lysates were immunoblotted with anti-Rab33B (#sc-271199) and anti-β-actin antibodies. (**C**) For immunofluorescence (IF) analyses, cells were fixed and permeabilized using methanol. This fixation method was chosen because the β-actin antibody is not reactive upon paraformaldehyde (PFA) fixation, as instructed by the supplier. Cells were stained with antibodies against calnexin (CNX), protein disulfide isomerase (PDI), golgin-97, and β-actin followed by staining with fluorophor-labeled antibodies. DNA staining is shown in blue. Scale bar (**C**): 10 µm.

The exocytosis of HBV viral particles depends on ESCRT functions, but it is less known whether the virus is exported via the secretory pathway or a MVB-guided exosomal route [8,9,10,11]. Therefore, we first assessed whether an interference with Rab33B functions may affect the constitutive secretion pathway. As a reporter, we used the HBV S envelope protein that is integrated into the ER membrane prior to assembly into intraluminal particles. These SVPs next traverse the Golgi complex and are finally released into the extracellular environment [6,15,17]. To study HBV S maturation and secretion, an expression construct carrying the S gene with a C-terminally tagged HA epitope (S.HA) was used. To perturb the Rab33B pathways in human hepatoma HuH-7 cells, Rab33B was depleted with a siRNA pool containing four different duplexes prior to transfection with S.HA. Cell lysates were subjected to Rab33B-specific immunoblotting which demonstrated an efficient knockdown of Rab33B (Figure 1A). The inspection of the intra- and extracellular S.HA levels revealed that the Rab33B knockdown did not interfere with the synthesis and secretion of S.HA (Figure 1A). Moreover, the typical *N*-glycosylation pattern of S.HA occurring in about half of the S molecules and the processing of the attached *N*-glycans within the Golgi complex did not differ between siControl (siCon)- and siRab33B-treated cells. The uncropped blots are shown in Figure S1. Hence, Rab33B is not essential for the assembly and release of HBV SVPs. In addition, we inspected Rab33B-knockdown cells for possible changes in cell morphology with special emphasis on the secretory apparatus, the cytoskeleton, and the plasma membrane (PM). Cells were transfected with siCon- or siRab33B-specific duplexes as above and subjected to immunofluorescence (IF) microscopy. Because the Rab33B-specific antibodies do not react in IF analyses, cell lysates of the same transfection trial were probed by Rab33B-specific WB that demonstrated an efficient Rab33B knockdown (Figure 1B). In parallel, cells were stained with antibodies against calnexin (CNX, a marker for the ER), protein disulfide isomerase (PDI, a marker for the intermediate compartment [IC]), golgin-97 (a marker for the Golgi complex), and β-actin that labels the actin skeleton, especially beneath the PM. As shown in Figure 1C, no substantial differences in the ER, IC, Golgi, and β-actin pattern were evident between the control and Rab33B-depleted cells. Hence, the loss of Rab33B does not compromise the overall cell morphology.

### 3.2. Role of Rab33B in HBV Assembly and Release

**Figure 2:**
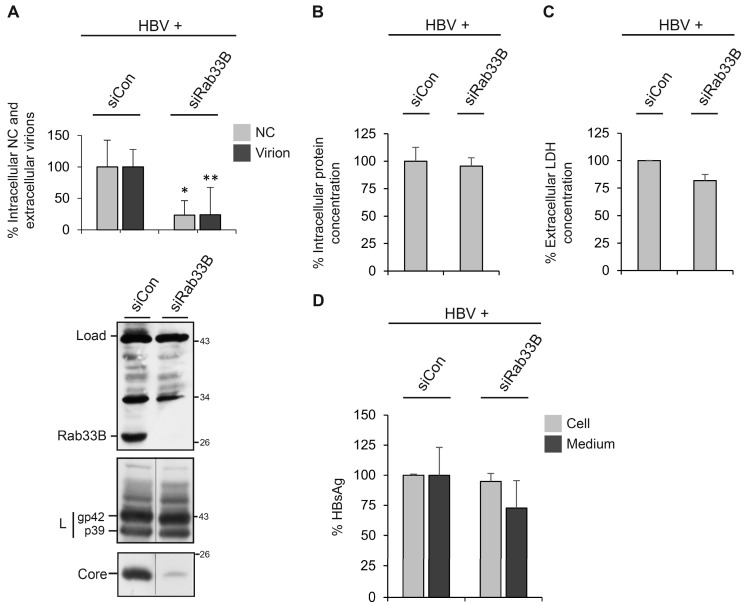
Depletion of Rab33B blocks HBV formation and release. (**A**) HuH-7 cells were treated with control small interfering RNA (siCon) or the Rab33B-specific small interfering RNA (siRab33B) pool for 48 h and were retransfected with with an HBV replicon construct (HBV). After 72 h, lysates and supernatants were harvested. To probe for Rab33B depletion, lysates were immunoblotted with an anti-Rab33B antibody (#6F4). Uniformity of sample loading is shown by an unspecific band stained by the antibody (Load). HBV release was detected by envelope-specific immunoprecipitation of supernatants and real-time PCR of the viral genomes (Virion). Nonenveloped cytoplasmic nucleocapsids (NC) were immunoprecipitated with anti-capsid antibodies and analyzed by PCR. PCR results were demonstrated in percent amount relative to the control-transfected cells. Error bars indicate the standard deviations from the mean of four experiments measured in duplicates. RNA interference (RNAi) effects on the expression of L and core were analyzed by specific WB of lysates prepared with 0.2% Triton X-100. The non-glycosylated (p39) and glycosylated forms (gp42) of L are depicted. Numbers to the right refer to molecular weight standards in kDa. * *P* < 0.05, ** *P* < 0.01 compared to control. (**B**) Total protein concentrations of lysates of siCon- or siRab33B-treated cells were determined using a Bradford assay and demonstrated in percent amount relative to the control cells (*n* = 2, mean ± SD). (**C**) To probe for cell lysis, supernatants of transfected cells were assayed for lactate dehydrogenase (LDH) activity (*n* = 2, mean ± SD). (**D**) To quantitate the levels of the HBV envelope proteins, lysates and supernatants of transfected cells were analyzed with a HBsAg-specific ELISA (*n* = 2, mean ± SD).

The hepatoma HuH-7 cell line is not susceptible to HBV infection because it expresses very low levels of the NTCP receptor [1]. Post-infection steps of the viral life cycle can be mimicked by transfection of cells with replication-competent HBV genomes. Notably, these cells can also not be reinfected with progeny virions and therefore provide a useful model to study the production and release of the virus rather than infection. To analyze whether Rab33B is involved in HBV morphogenesis, a siRNA-mediated knockdown was performed in HBV-replicating HuH-7 cells using the siRNA pool as above. After 48 h, cells were retransfected with the pHBV replicon, and cell lysates and supernatants were harvested after an additional 72 h. Lysates, prepared with the detergent Triton X-100, were subjected to Rab33B-specific WB which showed an almost complete lack of Rab33B expression (Figure 2A). The production and egress of HBV particles was determined by immunocapture of intracellular NC and extracellular virions with capsid- or envelope-specific antibodies, respectively, followed by particle disruption and real-time PCR measurement of the number of HBV genomes. As shown in Figure 2A, the knockdown of Rab33B significantly decreased both the amounts of intracellular NC and extracellular virions, as compared to siCon-treated cells. The raw PCR values are outlined in Figure S2. To gain insights into the underlying mechanisms, lysates were probed for the expression and stability profiles of the viral envelope and core proteins by WB. The intracellular steady-state levels of the L envelope protein, synthesized in non-glycosylated p39 and single-glycosylated gp42 forms, was largely unaffected, irrespective of whether Rab33B was depleted or not (Figure 2A). The uncropped blots are shown in Figure S3. Against expectations, the level of core was dramatically reduced in Rab33B-knockdown cells (Figure 2A). To rule out the possibility that the applied RNAi strategy might be harmful for the cells, total protein concentrations of cell lysates and LDH activity of cell supernatants were determined. Thereby, no differences in protein expression (Figure 2B) or evidence of cell damage (Figure 2C) could be observed in the Rab33B-depleted cells. Corresponding results were obtained when the lysates and supernatants were assayed with a quantitative HBsAg ELISA that measures the amounts of all three HBV envelope proteins. Consistent with the L-specific WB shown in Figure 2A, the depletion of Rab33B did not affect the intracellular concentration of the envelope proteins (Figure 2D). HBsAg reactivity, however, declined in supernatants of Rab33B-knockdown cells as compared to control cells (Figure 2D). This decline was less pronounced as compared to the reduction in virion release upon Rab33B inactivation (see Figure 2A), likely because the ELISA measures both virions and SVPs. From these data we conclude that the formation, trafficking, and/or release of HBV virions, but not of SVPs, requires the assistance of Rab33B. 

**Figure 3:**
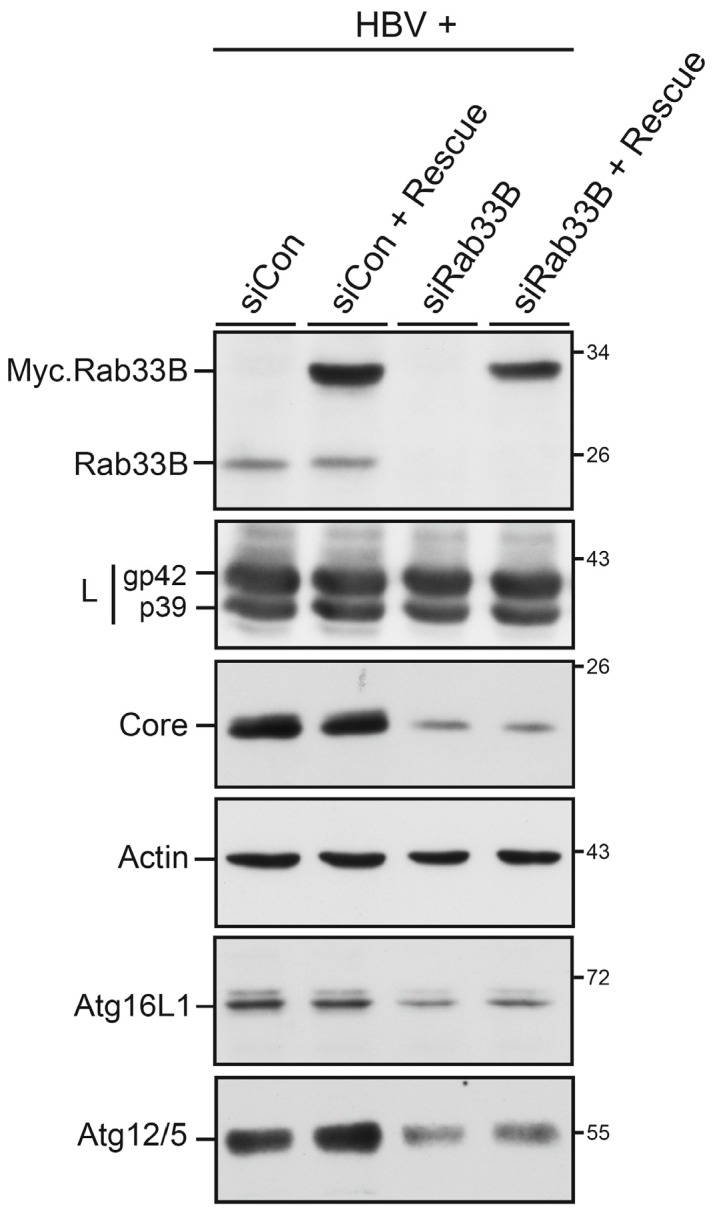
Effects of Rab33B depletion and reconstitution. HuH-7 cells were treated with control small interfering RNA (siCon) or the Rab33B-specific small interfering RNA (siRab33B) pool for 48 h and were retransfected for 72 h with HBV in the absence or presence of a mouse Myc-tagged Rab33B rescue construct (Rescue) at a 2:0.8 DNA weight ratio, respectively. Cell lysates were assayed by Rab33B-, L envelope protein-, Core-, β-actin-, Atg16L1-, and Atg5-specific immunoblotting. Experiments were done in duplicate and representative images are shown. Numbers to the right refer to molecular weight standards in kDa.

We next wondered whether the Rab33B-knockdown phenotype could be rescued by reexpression of a siRNA-resistant Rab33B construct. HuH-7 cells were treated with siCon- or siRab33B-specific duplexes and cotransfected with pHBV and an attenuated expression vector encoding Myc-tagged mouse Rab33B. Immunoblotting of cell lysates demonstrated efficient depletion of endogenous Rab33B and expression of exogenous Rab33B that showed a higher molecular weight due to the Myc-tag (Figure 3). However, upon re-expression of Rab33B, the HBV expression pattern did not change, as the levels of core remained reduced (Figure 3). Consequently, the decline in intracellular NC and extracellular virions induced by Rab33B silencing could not be recovered (data not shown). Because human and mouse Rab33B proteins are strongly conserved, we excluded species-specific effects for the lack of recovery. Rather, we inspected cells for so-called “codepletion phenomena”, i.e., whether the Rab33B-specific siRNA might simultaneously suppress protein levels of partner proteins, like the autophagic Atg5/12/16L1 complex. Often, the knockdown of individual subunits of protein complexes triggers the breakdown of the entire complex because the proper stoichiometry of complex partners and hence complex integrity are disturbed [10,11]. Indeed, as shown by Atg16L1- and Atg5-specific WB, the depletion of Rab33B led to codepletion of its Atg5/12/16L1 effector that could not be recovered upon sole Rab33B reexpression (Figure 3). Hence, Rab33B acts seemingly in conjunction with its autophagic effector to promote HBV propagation.

**Figure 4:**
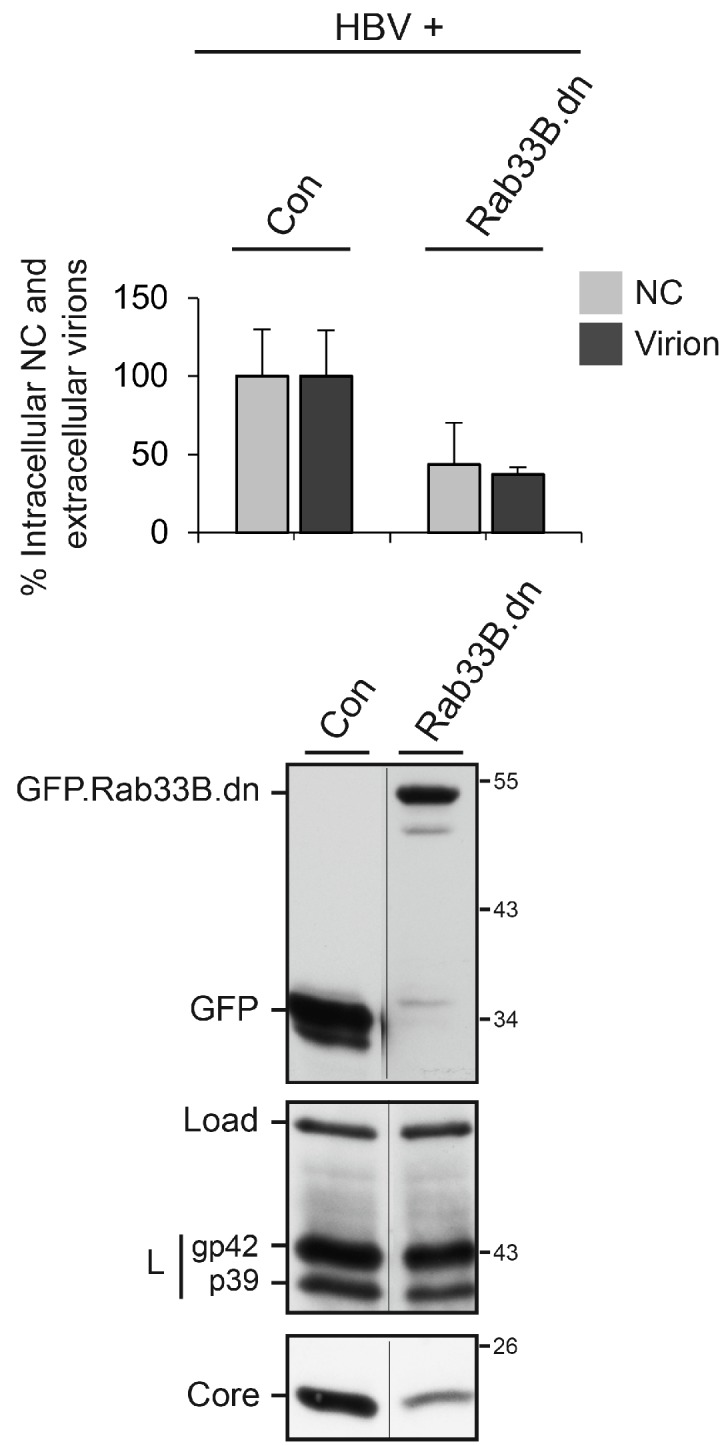
Overexpression of Rab33B.dn blocks HBV formation and release. HuH-7 cells were cotransfected with the HBV replicon and a green fluorescent protein (GFP)-encoding plasmid (Con) or a GFP-tagged Rab33B dominant negative (Rab33B.dn) mutant at a 1:3 DNA weight ratio, respectively. Three days post-transfection, cellular supernatants and cytoplasmic extracts were harvested and processed exactly as described in the legend of Figure 2A. In contrast, lysates were probed by GFP-specific WB. PCR results were demonstrated in the percent amount relative to the cells cotransfected with GFP. Error bars indicate the standard deviations from the mean of two experiments measured in duplicates. Numbers to the right refer to the molecular weight standards in kDa.

Similar results were obtained when endogenous Rab33B was inactivated by ectopic overexpression of its GTP binding-deficient mutant that is known to act in a dominant negative manner [20]. In this setting, HuH-7 cells were cotransfected with the HBV replicon in combination with either GFP or a GFP-tagged Rab33B.dn construct. Three days post-transfection, cell lysates and supernatants were harvested and assayed as above. The coexpression of GFP-tagged Rab33B.dn potently decreased the amounts of intracellular NC and extracellular virions (Figure 4). The raw PCR values are outlined in Figure S4. Again, the inspection of the HBV protein levels indicated that L expression was not affected, while the amount of core was strongly diminished upon overexpression of Rab33B.dn (Figure 4). The uncropped blots are shown in Figure S5. Given that the overexpression of the Rab33B.dn mutant phenocopied the effect of the Rab33B knockdown, we conclude that HBV formation, trafficking, and/or release require the assistance of catalytic active Rab33B. 

### 3.3. HBV Replication Upregulates Rab33B Gene Expression

**Figure 5:**
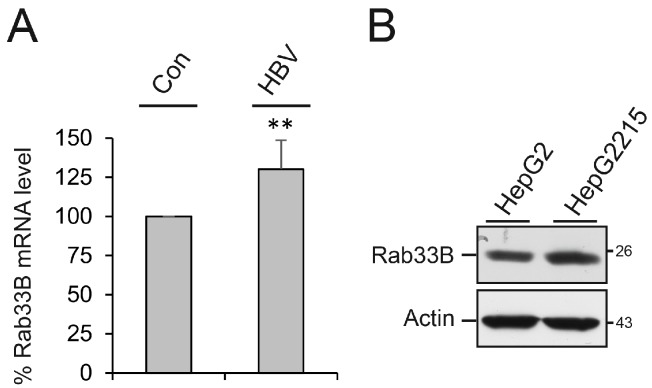
HBV upregulates Rab33B gene expression. (**A**) HuH-7 cells were mock-transfected (Con) or transfected with the pHBV replicon. After 72 h, total mRNAs were isolated, reverse transcribed, and used for quantitative reverse transcription PCR (qRT-PCR) reactions. Fold changes in Rab33B gene expression were calculated by comparing mRNA levels in control- or pHBV–transfected cells using β-actin as an endogenous reference gene. Error bars indicate the standard deviations from the mean of five experiments measured in duplicates. ** *P* < 0.01 compared to the control. (**B**) Equal cell numbers of HepG2 and HepG2215 cells were lysed and subjected to Rab33B-specific WB (#sc-271199). To control gel loading, the same blot was reprobed with anti-β-actin antibodies. Numbers to the right refer to the molecular weight standards in kDa.

Since Rab33B-guided processes are pivotal for the HBV replication cycle, we next investigated whether HBV replication had an effect on cellular Rab33B gene expression. HuH-7 cells were transiently transfected with an empty plasmid backbone or the pHBV replicon for three days. For gene expression profiling, total mRNA was isolated and subjected to quantitative reverse transcription-PCR (qRT-PCR) with Rab33B-specific primers. As shown in Figure 5A, HBV replication upregulated Rab33B transcription by about 25% as compared to control cells. This upregulation, however, could not be observed at the protein level using the less sensitive immunoblotting technique. This is likely due to the degree of transfection, as only about 20% of the HuH-7 cells received DNA in a lipofection-based transfection assay [31]. To circumvent this limitation, the Rab33B protein levels were comparatively analyzed in stably HBV-expressing HepG2215 and parental HepG2 cell lines. As illustrated in Figure 5B, the amount of Rab33B was clearly increased in HepG2215 cells as compared to control cells.

### 3.4. Effects of Rab33B Inactivation on HBV Transcription, Protein Expression, and Solubility

**Figure 6:**
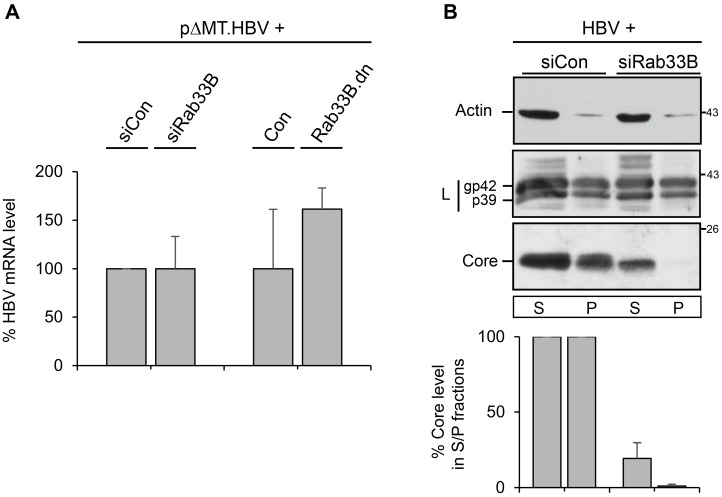
Effects of Rab33B inactivation on HBV transcription, HBV protein expression, and solubility. (**A**) For HBV gene expression profiling, HuH-7 cells were treated with control small interfering RNA (siCon) or the Rab33B-specific small interfering (siRab33B) pool and were retransfected with the pHBV∆HP replicon. In parallel, cells were cotransfected with pHBV∆HP and a GFP plasmid (Con) or the GFP-tagged Rab33B.dn mutant. For qRT-PCR, total mRNAs were isolated, reverse transcribed, and applied to PCR reactions using HBV-specific primer sets. Error bars indicate the standard deviations from the mean of two experiments measured in duplicates. (**B**) Rab33B knockdown reduces detergent-soluble and detergent-insoluble core fractions without affecting L protein levels. HuH-7 cells treated with siCon- or siRab33B-specific duplexes were retransfected with pHBV. Seventy-two h after DNA transfection, cell lysates were prepared with radioimmune precipitation (RIPA) buffer, separated into detergent-soluble (soluble (S)) and -insoluble fractions (pellet (P)), and analyzed by WB for the presence of L and core proteins and endogenous β-actin. The levels of core proteins were quantified by densitometric analysis of Western blots and were demonstrated in percent amount relative to the control cells (*n* = 4, mean ± SD).

To gain insights how Rab33B inactivation compromised the synthesis/stability of core and hence HBV replication, we first analyzed the HBV transcription profile in Rab33B-knockdown and Rab33B.dn-expressing cells. The pHBV replicon used thus far contains a 1.1× unit length HBV genome in which the foreign hMT promoter precedes the viral core/Pol promoter. Because this feature may hamper the transcriptional analyses, a modified HBV replicon (pHBV∆HP) with a deletion of the hMT promoter was used. In previous analyses, we could validate the functionality of this modified replicon [10]. To study HBV transcription in Rab33B-impaired cells, total mRNA was extracted and measured by qRT-PCR using primers targeting the HBV pgRNA, and the core/pol- and envelope-specific transcripts. Thereby, we did not detect gross changes in the synthesis and stability of the HBV transcripts (Figure 6A), indicating that neither the Rab33B knockdown nor Rab33B.dn overexpression hindered HBV transcription.

Next, we focused on the solubility profile of the cell-associated core. In order to detect the formation of progeny NC by immunocapture and PCR analysis, cell lysis with mild detergents (0.2% Triton X-100) was applied (see Figure 2). Under these conditions, less soluble proteins could get lost. To bypass this issue, lysis of transfected cells was performed in more stringent buffer (RIPA) including the denaturing detergent SDS, and lysates were separated into detergent-soluble and -insoluble fractions. As above, the L-specific WB did not reveal major differences in the levels of detergent-soluble and -insoluble L protein in siCon- or siRab33B-treated cells (Figure 6B). In contrast, the amounts of the detergent-soluble and -insoluble core were strongly reduced in Rab33B-depleted cells (Figure 6B). To account for this, we assume that the loss of Rab33B might misdirect proper NC formation and trafficking and/or cause NC destabilization concomitant with total core protein reduction. 

### 3.5. Rab33B Knockdown Alters Intracellular Core/Capsid Distribution without Affecting the Envelope 

**Figure 7:**
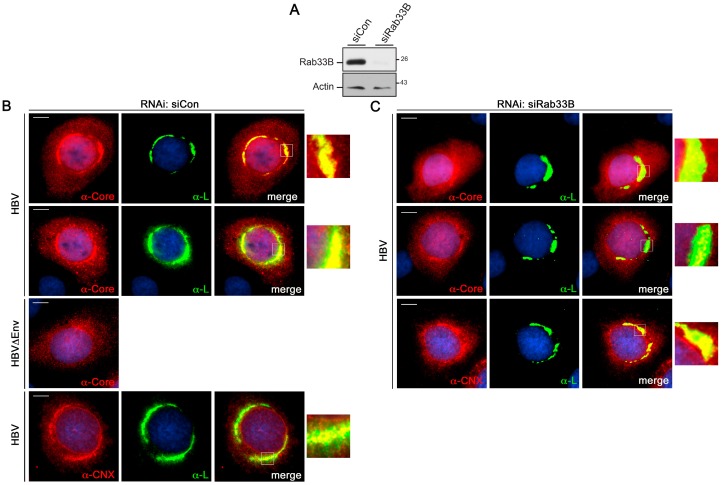
Effects of Rab33B inactivation on intracellular HBV protein distribution. For RNAi, HuH-7 cells were treated with control or Rab33B-specific siRNAs for two days and were retransfected for two days with pHBV or pHBV∆Env, as indicated to the left of the images. (**A**) To probe for depletion, cells were lysed and assayed by Rab33B- (#sc-271199) and β-actin-specific WB. (**B**,**C**) For IF, cells were fixed and permeablized with PFA and Triton X-100. Cells were immunostained with anti-core (Dako), anti-L (MA18/7), or anti-calnexin (CNX) antibodies followed by staining with AlexaFluor 546-conjugated anti-rabbit and AlexaFluor 488-conjugated anti-mouse antibodies. Core and CNX staining is in red, L staining in green, and DNA staining of the nuclei is in blue. Scale bar: 10 µm. Merge images are shown in the right panels. Outlined areas are shown at larger magnifications.

This prompted us to study the fate of the HBV proteins on a single cell level. For IF analyses, HuH-7 cells were treated with siRNAs, retransfected with pHBV, fixed with PFA, and permeabilized with Triton X-100. The depletion efficacy was assessed by Rab33B-specific WB as above (Figure 7A). Cells were co-stained with L-specific antibodies and an antibody (Dako) known to recognize core monomers, capsids, and the pre-core protein. In siCon-treated cells, core yielded its typical diffuse staining dispersed throughout the cytoplasm with some nuclear labelling. Aside, a prominent perinuclear accumulation of core in a ring-like structure was detectable (Figure 7B). To ascertain that this structure mainly reflected core rather than the ER-associated pre-core protein, cells were transfected with a pHBV∆Env mutant replicon that is identical to pHBV, save for its inability to drive envelope protein synthesis. As exemplified in Figure 7B, the ring-like, perinuclear accumulation of core was absent in these cells, implicating that it mirrored core, rather than pre-core, recruited by the envelope proteins in wild-type cells. L revealed a confined perinuclear, ER-like staining with enrichment in juxtanuclear regions where it intensely colocalized with core in pHBV-expressing cells (Figure 7B). The L/core colocalizing area partly overlapped with ER structures, as inferred by the costaining pattern of L and calnexin. Upon Rab33B depletion, the staining pattern of core, but not of L, changed. L retained its restricted peri/juxtanuclear localization where it partly colocalized with calnexin. Consistent with our biochemical data, core protein levels were found to be reduced in Rab33B knockdown cells, where images were captured with automatic, i.e., optimal-adjusted exposure times. Even so, core barely accumulated in the perinuclear, ring-like structures, concomitant with a significant reduction in the degree of core/L colocalization (Figure 7C). These data implicate that the lack of Rab33B may block or misdirect proper core trafficking to NC assembly/envelopment sites, thereby rendering core/NC sensitive to destabilization and destruction. 

### 3.6. HBV Core Associates with Membranes via its Arginine-Rich CTD Domain

**Figure 8:**
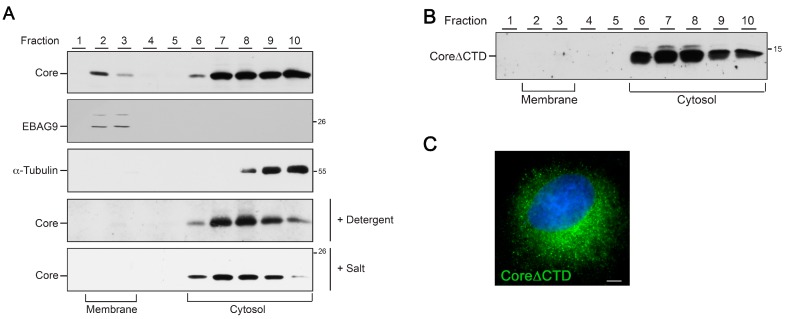
Core associates with membranes via its C-terminal arginine-rich domain. (**A**) HuH-7 cells were transiently transfected with pCore for three days. Cells were lysed by sonication and membrane flotation was performed. Ten fractions were collected from the top of the gradient and analyzed by core-specific WB. The blots were reprobed with antibodies specific for α-tubulin and estrogen receptor binding site associated antigen 9 (EBAG9) to determine the cytosolic or membrane fractions, respectively. Experiments were done in triplicate and representative images are shown. In the panels depicted with “+ Detergent” or “+ Salt”, the postnuclear supernatants were adjusted with 0.5% Triton X-100 or 150 mM NaCl, respectively, prior to membrane flotation. Numbers to the right refer to the molecular weight standards in kDa. (**B**) Cells were transfected with the Core∆CTD mutant lacking CTD and assayed as above. (**C**) HuH-7 cells expressing Core∆CTD were immunostained with a capsid-specific antibody (#3HB17) followed by staining with AlexaFluor 488-conjugated anti-mouse antibodies. DNA staining of the nuclei is in blue. Scale bar (**C**): 10 µm.

A prerequisite for the budding of enveloped virus particles is the assembly of all viral components at a conjoint cellular membrane. In the case of many retroviruses, membrane targeting of the viral gag is mediated through N-terminal acylation and basic aa patches of the matrix domain [32,33]. Since the HBV core protein does not contain a typical matrix domain, its targeting to the budding site remains enigmatic. To study membrane association of core, membrane flotation analyses were performed. HBV core/capsids are known to interact with the membrane-embedded viral envelope proteins that may complicate the interpretation of the results. Therefore, we first studied core membrane association in the absence of the envelope proteins by using a construct encoding only core (pCore; devoid of the pre-core region). Transfected HuH-7 cells were subjected to membrane flotation and flotation fractions were analyzed by WB. For controls, the cellular EBAG9 protein served as a reference for a membrane-associated protein, while α-tubulin was used as a marker for a soluble protein. As expected, the α-tubulin signal was found in the high density-phase of the gradient, whereas EBAG9 was only detected in low density-phases (Figure 8A). Importantly, the core protein was found to partition between the soluble and membrane fractions, indicating that a fraction of core is targeted to membranes (Figure 8A). As a formal proof, lysates were treated with detergent prior to membrane flotation analysis. Under these conditions, core was undetectable in the low density-phase fractions. While screening for optimal binding conditions, we found that core membrane association is sensitive to increasing salt concentrations (Figure 8A), implicating that electrostatic interactions may be involved. This prompted us to analyze whether the arginine-rich, CTD of core may be a determinant for membrane association. The analysis of a core mutant lacking CTD demonstrated a complete defect in membrane association (Figure 8B). Hence, CTD of core mediates not only the packaging of the viral pgRNA/Pol complex [2,3] but also acts as a membrane-binding module. Previous studies have shown that the CTD-deficient mutant is competent to assemble into typical capsids [34,35,36]. For verification, cells expressing Core∆CTD were subjected to immunofluorescence microscopy using the capsid-specific antibody that clearly recognized the core mutant (Figure 8C). From these data we infer that the membrane association of core is not mandatory for capsid assembly per se.

### 3.7. Rab33B Knockdown Impairs Membrane Association of HBV Core

**Figure 9:**
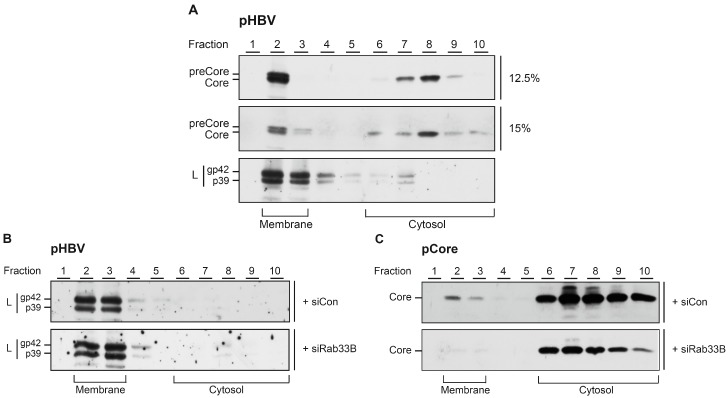
Core associates with membranes in a Rab33B-dependent manner. (**A**) HuH-7 cells were transfected with pHBV for 72 h and were subjected to density flotation analyses. Gradients were fractioned from the top, fractions were run on 12.5 or 15% sodium dodecyl sulphate (SDS) polyacrylamide gels, and analyzed by core- or L-specific WB. Assays were done in duplicate and representative images are shown. Membrane and cytosolic fractions are depicted. Note that the 22 kDa pre-core and 21 kDa core proteins comigrate in form of double bands. (**B**) HuH-7 cells treated with control or Rab33B-specific siRNAs were retransfected with pHBV. Cells were lysed by sonication followed by membrane flotation and L-specific WB. Membrane and cytosolic fractions are depicted. (**C**) SiRNA-treated cells were transfected with pCore and subjected to membrane flotation analyses and core-specific WB.

In parallel, core membrane association was analyzed in the context of an ongoing HBV replication. HuH-7 cells were transfected with pHBV and analyzed by membrane flotation as above. Under these conditions, core was again detectable in the membrane fractions (Figure 9A). Of note, the closely related pre-core protein that is synthesized as a 22 kDa precursor protein at the ER membrane comigrates with the 21 kDa core protein in the membrane fractions. To improve the separation of these bands, samples were run on either 12.5 or 15% SDS–PAGE (Figure 9A). Upon reprobing of the blot with envelope-specific antibodies, the transmembrane L protein was found to be enriched in the membrane fractions (Figure 9A), thus confirming the effective separation of cytosolic and membrane fractions under the applied assay conditions. Together, these results indicate that core is able to associate with membranes in the presence and absence of the viral envelope. 

Like all Rab proteins, Rab33B acts as a molecular switch, cycling between a soluble and membrane-bound form, in order to regulate membrane traffic [19]. Accordingly, we reasoned to study whether the membrane association of core may be conjoint with Rab33B. HuH-7 cells were treated with siCon- or siRab33B-specific duplexes prior to transfection with pHBV. Subsequent membrane flotation analyses revealed that the distribution of L and its enrichment in the membrane fractions were not affected by deficit Rab33B (Figure 9B). Due to the Rab33B RNAi-induced downregulation of core in HBV-replicating cells, we failed to unequivocally analyze the distribution of core under these conditions (data not shown). For less understood reasons, the Rab33B RNAi-induced core protein reduction is less pronounced upon sole expression of core [18] (see also the discussion). We therefore studied membrane association of core synthesized in the absence of the other HBV proteins, especially of the pre-core protein. Subsequent membrane flotation analyses revealed that the pool of membrane-associated core was diminished in Rab33B-knockdown cells (Figure 9C). For quantification, cell extracts were assayed by a pre-core/core-specific ELISA prior to loading equal core amounts to flotation gradients. ELISA measurements of the fractions showed that the depletion of Rab33B reduced core membrane association by 49.3 ± 6.3% (*n* = 2) as compared to siCon-treated cells.

## 4. Discussion

In this study, we demonstrated the importance of the cellular Rab33B GTPase in the production of infectious HBV. Upon in vitro replication in permissive hepatoma cells, the inactivation of Rab33B led to a decline in the levels of extracelluar virions and intracellular NC, likely as a consequence of defects in intracellular virus traffic events. The overexpression of an inactive, GDP-restricted Rab33B mutant phenocopied the effects of deficit Rab33B, implicating that effector proteins may be involved. Rab33b is a known regulator of Golgi retrograde trafficking, but also plays roles in autophagy processes, as it uses the autophagic Atg5/12/16 complex as an effector [20,21,22].

Given the role of Rab33B in Golgi homeostasis, we first assessed the fate of the HBV envelope proteins in knockdown cells. By using the S envelope protein as a surrogate marker of the constitutive secretory pathway, we observed that deficit Rab33B had no impacts on the synthesis of S, its modification with *N*-glycans and extracellular release in form of SVPs. Consistent with this, the anterograde transport of a G protein mutant of vesicular stomatitis virus has been reported to precede normally in the Rab33B-depleted Golgi apparatus [21]. In addition, we found that the synthesis and modification of the L envelope protein, its enrichment in subcellular membrane fractions, and its colocalization with ER markers, were not compromised in Rab33B-inactivated cells, implicating the the viral envelope is not the main target of Rab33B intervention.

By analyzing the effects of deficit Rab33B on virus replication steps, we found that it did not interfere with the transcription of the HBV genome, but dramatically impeded the synthesis, assembly, and/or stability of core/capsids. In particular, the protein levels of core, its intracellular distribution, and membrane targeting were strongly altered in Rab33B-knockdown cells. As a consequence, NC assembly and/or NC envelopment and thus virus secretion were blocked in an Rab33B activity-dependent manner. Thus far, HBV capsid assembly had mainly been viewed as a spontaneously occurring process of self-assembly, dependent only on the presence of capsid protein subunits themselves [2,3,4]. However, recent reports implicate that viral capsid assembly is an active process executed by host factors that may act as assembly machines [37]. In the case of HBV capsids, the host serine–arginine protein kinase had been reported to act like a molecular chaperone to prevent the core protein from assembling at the wrong time and place [38]. On the contrary, members of the cellular heat shock protein family 40 were shown to negatively regulate capsid assembly through destabilization and degradation of the core protein [39]. Besides, HBV capsid assembly can be modulated by chemical compounds, like heteroaryldihydropyrimidines (HAP), that are considered as promising non-nucleos(t)ide HBV replication inhibitors. The first HAP compound Bay 41–4109 had been shown to inhibit core particle formation as the primary event concomitant with an increased proteasomal degradation of core protein as a consequence [40]. According to the results shown herein, Rab33B is another important player in proper HBV capsid processing. The abrogation of Rab33B-guided assembly and/or trafficking events may trap core/NC to locations where they become unavailable for the generation of progeny virions and thus are prone to degradation, a phenotype reminiscent for the action of HAPs.

Genetic approaches led to the identification of short linear domains in the envelope proteins and two areas on the capsid surface where point mutations block NC envelopment [29,41,42]. It has been suggested that these domains are involved in envelope/capsid interactions driving the budding process [5], thereby possibly sparing membrane association of the core/capsid. By addressing this point, we observed that core clearly binds membranes even in the absence of the viral envelope. Membrane association of core is mediated by its arginine-rich CTD and hence may involve electrostatic interactions with negatively-charged lipids. In this respect, the HBV core shares features of retroviral gag proteins that employ basic patches of their matrix domains in conjunction with N-terminal acylation for membrane targeting [33]. The multiple roles of CTD played in the HBV life cycle [4,35] can thus be expanded for a function as a membrane-binding module. Aside from the basic charge, the phosphorylation of core within CTD may contribute to membrane association. In case of the related duck HBV (DHBV) core protein, it had been shown that capsid dephosphorylation leads to the exposure of a membrane-binding signal as a crucial step for the budding of virus particles [43]. Consistent with previous reports [4,34,36], Core∆CTD is competent for capsid assembly, at least under conditions of overexpression. We thus infer that membrane surfaces are not mandatory for capsid formation per se. Noteworthy, however, is that membrane requirements for the capsid and NC assembly reactions may differ, as NC formation encounters pgRNA/Pol complex packaging in the crowded cytoplasm. HBV may face this problem by directing NC assembly to membranes in order to raise the local concentration of the viral subunits. Such a strategy is used by many viruses that replicate in the cytoplasm and compartmentalize their assembly reactions in subcellular microenvironments in order to increase multiplication efficiency and to protect against host cell defenses [32,44].

The inactivation of Rab33B reduces the pool of membrane-bound HBV core/capsids, implicating a host-assisted membrane targeting mechanism. Like all Rab GTPases, Rab33B efficiently binds to membranes via its fatty acid moiety [19]. To examine whether Rab33B may piggyback core to membranes, we performed coimmunoprecipitation and GST pulldown assays. Thereby, however, we obtained no hints for a direct physical interaction between core and Rab33B. Rather, a link may be established by the autophagic Atg5/12/16 complex, a known effector of active Rab33B, that we previously identified as a core-interacting partner [18]. In support, the depletion of Atg5/12/16 likewise prevents core membrane association [31]. It is therefore conceivable that core may co-opt Rab33B via its bound Atg5/12/16 effector as a device for membrane binding. The intimate crosstalk between Rab33B and its effector was affirmed by our quite unexpected finding that Rab33B silencing led to codepletion of Atg16L1 and the Atg12/5 conjugate, likely as a consequence of complex destabilization. The decrease in core membrane association coincides with the block in NC formation, likely as a consequence of improper sorting and trafficking of core to NC assembly site(s) in Rab33B-knockdown cells. However, because it is less clear where the pgRNA/Pol complex enters the NC assembly pathway, we cannot formally exclude the possibility that the loss of Rab33B may affect pgRNA/Pol transport as well. Aside, it is equally possible that Rab33B may direct the trafficking of premade NC to sites of the final envelopment reaction. In favor of core/NC missorting events, we observed that the degree of colocalization between core and the envelope is largely reduced in Rab33B-knockdown cells. In the case of the *Paramyxoviridae* [45,46], *Orthomyxoviridae* [47], and *Bunyaviridae* [48] families, viral ribonucleoproteins (vRNPs) attach to Rab11-positive vesicles for trafficking to budding zones. How vRNPs attach to these vesicles is less clear, but the association between vRNPs and Rab11 seems to occur only with the Rab11 active form [46,47]. 

HBV is supposed to bud at intracellular membranes, but the budding site has not been unequivocally defined. Electron microscopy studies of HBV-infected cells and HBV-infected liver biopsies have shown that the virus assembles in large intracellular compartments that may resemble late endosomes or MVBs [49,50]. The ESCRT dependency of HBV is another clue for MVBs serving as HBV budding sites [8,9,10,11]. Because Rab33B has been linked to autophagosome formation, and autophagosomal structures can fuse with MVBs to form amphisomes [20,22], the Rab33B-assisted delivery of NC to the HBV budding site may also involve autophagy-related membranous structures. 

Notably, our previous work aimed at characterizing the role of Rab33B in HBV naked capsid release, the downregulation of core/capsids induced by deficit Rab33B was less prominent [18] (see also Figure 9C). To account for the different phenotypes, we assume that autophagy pathway functions may be involved. The sole expression of core does not induce autophagy [18], while the expression of an HBV replicon construct does [26]. HBV has been reported to enhance and use autophagy for its replication, but the underlying mechanisms are a matter of debate. Upon pharmaceutical or genetic inhibition of autophagy, one set of experiments indicates that NC maturation was found to be impaired in HBV transgenic mice, whereas in HBV-replicating cell lines the envelopment of NC was primarily inhibited [23,24,25,26]. In agreement, however, most studies reported that HBV stimulates autophagy without ending up in lysosomes [23,24,25,26]. Possibly, this is mediated by the HBV X protein that has been shown to inhibit autophagic degradation, if overexpressed [51]. Therefore, the induction of autophagy concomitant with the inhibition of lysosomal degradation in HBV-replicating cells may promote proteasomal destruction of core/capsids that are missorted in Rab33B-depleted cells. 

The important role of Rab33B in the HBV life cycle is substantiated by its upregulation upon virus replication. Interestingly, the replication and secretion of the hepatitis C virus (HCV) also depend on the action of Rab33B [52]. Like HBV, HCV upregulates Rab33B expression, likely to facilitate the accumulation of viral proteins during the formation of membranous replication factories [52]. Increased expression of Rab33B may be required for HCV, as it is involved in autophagy, which has been shown to be important for HCV propagation [53]. Given that both HBV and HCV increase Rab33B protein levels and depend on autophagy pathway functions for benefits, it is tempting to speculate that Rab33B may play a general role in autophagy-assisted virus assembly reactions. The elucidation of the precise role of Rab33B, possibly in cooperation with the autophagy network, in virus infections will be an important step next forward.

## Supplementary Materials

The following are available online at www.mdpi.com/1999-4915/9/5/157/s1, Figure S1: Depletion of Rab33B does not affect HBV SVP release, Figure S2: Depletion of Rab33B blocks HBV formation and release, Figure S3: Depletion of Rab33B reduces protein levels of core, but not of L, Figure S4: Overexpression of Rab33B.dn reduces protein levels of core, but not of L, Figure S5: Overexpression of Rab33B.dn blocks HBV formation and release.

## Abbreviations

The following abbreviations are used in this manuscript:

RNAi	RNA interference
HBV	hepatitis B virus
NTCP	taurocholate co-transporting polypeptide
dsDNA	double-stranded DNA
Pol	polymerase
NC	nucleocapsid
cccDNA	covalently closed circular DNA
pgRNA	pregenomic RNA
aa	amino acid
CTD	C-terminal domain
ER	endoplasmic reticulum
ESCRT	endosomal sorting complex required for transport
MVB	multivesicular body
SVP	subviral particles
Atg	autophagy
hMT	human metallothionein
CMV	cytomegalovirus
SV40	Simian virus 40
HA	hemagglutinin
dn	dominant negative
EGFP	enhanced green fluorescent protein
GST	glutathione *S*-transferase
CNX	calnexin
EBAG9	anti-estrogen receptor binding site associated antigen
PDI	protein disulfide isomerase
WB	Western blotting
HBsAg	hepatitis B surface antigen
LDH	lactate dehydrogenase
RIPA	radioimmune precipitation buffer
EDTA	ethylenediaminetetraacetic acid
PFA	paraformaldehyde
EGTA	ethylene glycol-bis(β-aminoethyl ether)-*N*,*N*,*N*',*N*'-tetraacetic acid
PBS	phosphate-buffered saline
HEPES	hydroxyethyl piperazineethanesulfonic acid
DB	dilution buffer
siCon	siControl
PM	plasma membrane
IF	immunofluorescence
IC	intermediate compartment
kDa	Kilodalton
qRT-PCR	quantitative reverse transcription-PCR
DHBV	duck HBV
HAP	heteroaryldihydropyrimidine
vRNPs	viral ribonucleoproteins
HCV	hepatitis C virus
