# Supplementary material for: Rab33B Controls Hepatitis B Virus Assembly by Regulating Core Membrane Association and Nucleocapsid Processing
Source: Viruses. 2017 Jun 21;9(6):157. doi: 10.3390/v9060157 (PMC5490832; doi:10.3390/v9060157)
Supplement: Supplementary file 1 [file viruses-09-00157-s001.pdf]

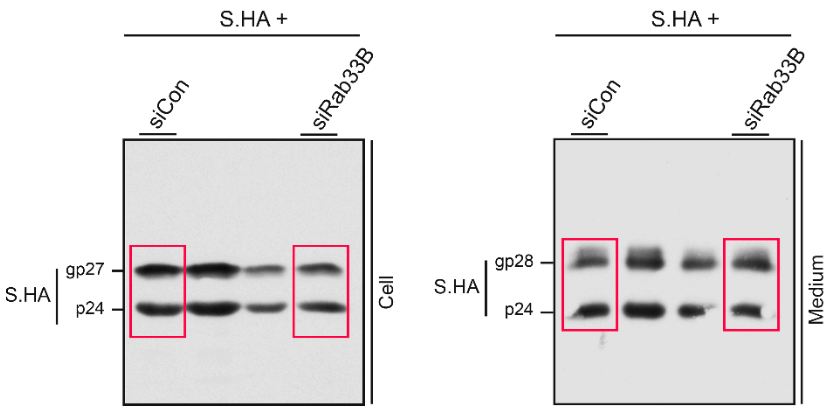

Figure S1. Depletion of Rab33B does not affect HBV SVP release. HuH-7 cells were treated with control small interfering RNA (siCon) or small interfering RNA duplexes directed against Rab33B. After 48 h, cells were retransfected with an HA-tagged version of the HBV S envelope gene (S.HA), and lysates and supernatants were harvested 72 h later. Synthesis (Cell) and secretion (Medium) of S.HA were probed by HA-specific immunoblotting. The uncropped blots are shown; red rectangles indicate the sections of the blots that are presented in Figure 1A. The non-glycosylated (p24) and glycosylated forms (gp27, gp28) of S.HA are indicated.

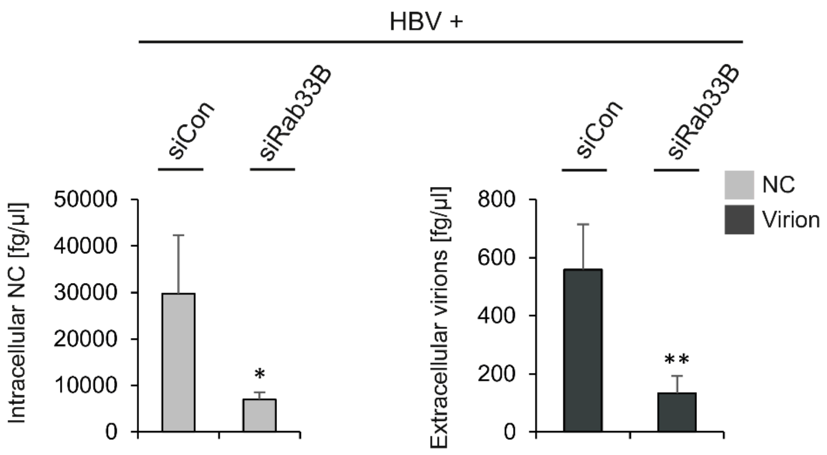

Figure S2. Depletion of Rab33B blocks HBV formation and release. HuH-7 cells were treated with control small interfering RNA (siCon) or the Rab33B-specific small interfering RNA (siRab33B) pool for 48 h and were retransfected with HBV. After 72 h, lysates and supernatants were harvested. HBV release was detected by envelope-specific immunoprecipitation of supernatants and real-time PCR of the viral genomes (Virion). Non-enveloped cytoplasmic nucleocapsids (NC) were immunoprecipitated with anti-capsid antibodies and analyzed by PCR. Raw PCR values indicate the concentration (fg/µL) of HBV genomes. * $P < 0.05$, ** $P < 0.01$ compared to control.

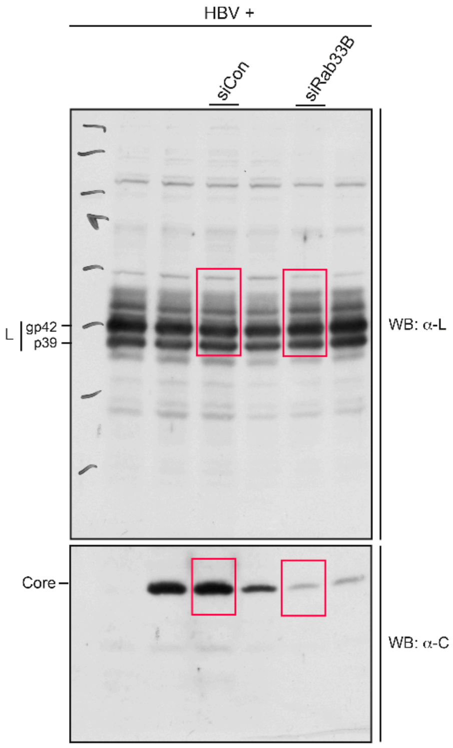

Figure S3. Depletion of Rab33B reduces protein levels of core, but not of L. HuH-7 cells were treated with siRNAs and transfected with HBV, exactly as described in Figure S2. RNAi effects on the expression of L and core were analyzed by immunoblotting with anti-L (top) or anti-core (bottom) of lysates. The uncropped blots are shown; red rectangles indicate the sections of the blots that are presented in Figure 2A. The non-glycosylated (p39) and glycosylated (gp42) forms of L are indicated.

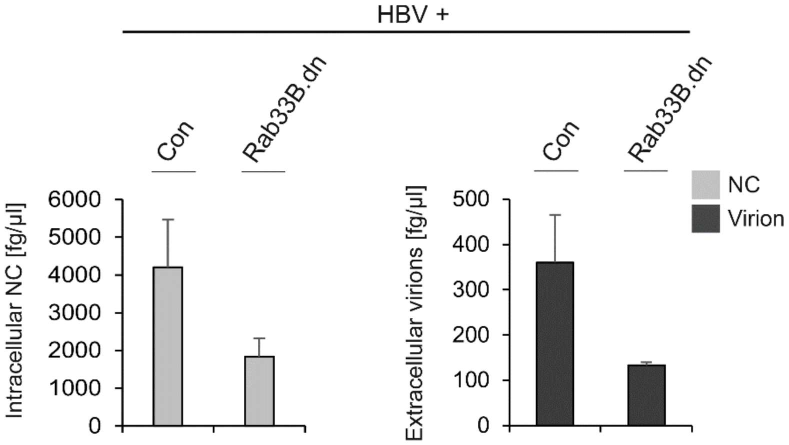

Figure S4. Overexpression of Rab33B.dn blocks HBV formation and release. HuH-7 cells were cotransfected with the HBV replicon and a GFP-encoding plasmid (Con) or a GFP-tagged Rab33B.dn mutant. After transient expression for three days, cellular supernatants and cytoplasmic extracts were harvested. Extracellular virions and intracellular nucleocapsids (NC) were immunoprecipitated with L- and core-specific antibodies, respectively, and were analyzed by real-time PCR. Raw PCR values indicate the concentration (fg/μL) of HBV genomes.

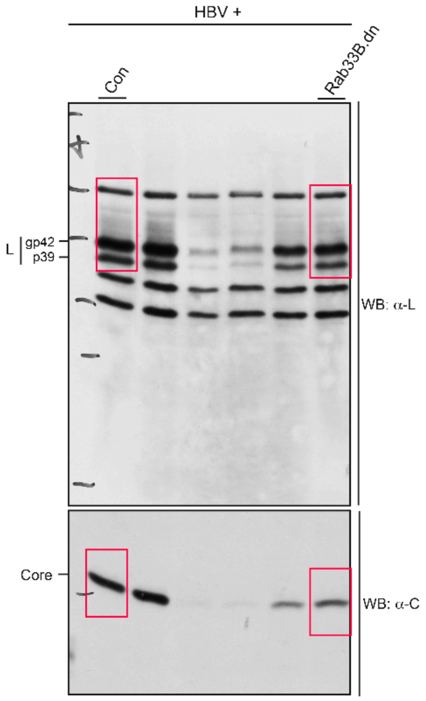

Figure S5. Overexpression of Rab33B.dn reduces protein levels of core, but not of L. HuH-7 cells were cotransfected with the HBV replicon and a GFP-encoding plasmid (Con) or a GFP-tagged Rab33B.dn mutant. Three days post-transfection, cellular supernatants and cytoplasmic extracts were harvested. RNAi effects on the expression of L and core were analyzed by immunoblotting with anti-L (top) or anti-core (bottom) of lysates. The uncropped blots are shown; red rectangles indicate the sections of the blots that are presented in Figure 4. The non-glycosylated (p39) and glycosylated (gp42) forms of L are indicated.
